# Autonomous Quality Control of Joint Orientation Measured with Inertial Sensors

**DOI:** 10.3390/s16071037

**Published:** 2016-07-05

**Authors:** Karina Lebel, Patrick Boissy, Hung Nguyen, Christian Duval

**Affiliations:** 1 Faculty of Medicine and Health Sciences, Orthopedic service, department of surgery, Université de Sherbrooke, Sherbrooke, QC J1H 5N4, Canada; Karina.Lebel@usherbrooke.ca; 2 Research Center on Aging, Sherbrooke, QC J1H 4C4, Canada; 3 Interdisciplinary Institute for Technological Innovation (3IT), Université de Sherbrooke, Sherbrooke, QC J1K 0A5, Canada; 4 Département des Sciences de l’activité Physique, Université du Québec à Montréal, Montreal, QC H2X 1Y4, Canada; hpnguyen@utexas.edu (H.N.); duval.christian@uqam.ca (C.D.); 5 Centre de Recherche Institut Universitaire de Gériatrie de Montréal, Montreal, QC H3W 1W4, Canada

**Keywords:** AHRS, IMU, MIMU, MARG, inertial sensors, attitude and heading reference system, 3D orientation tracking, joint orientation, artificial neural network, inertial motion capture, quality control

## Abstract

Clinical mobility assessment is traditionally performed in laboratories using complex and expensive equipment. The low accessibility to such equipment, combined with the emerging trend to assess mobility in a free-living environment, creates a need for body-worn sensors (e.g., inertial measurement units—IMUs) that are capable of measuring the complexity in motor performance using meaningful measurements, such as joint orientation. However, accuracy of joint orientation estimates using IMUs may be affected by environment, the joint tracked, type of motion performed and velocity. This study investigates a quality control (QC) process to assess the quality of orientation data based on features extracted from the raw inertial sensors’ signals. Joint orientation (trunk, hip, knee, ankle) of twenty participants was acquired by an optical motion capture system and IMUs during a variety of tasks (sit, sit-to-stand transition, walking, turning) performed under varying conditions (speed, environment). An artificial neural network was used to classify good and bad sequences of joint orientation with a sensitivity and a specificity above 83%. This study confirms the possibility to perform QC on IMU joint orientation data based on raw signal features. This innovative QC approach may be of particular interest in a big data context, such as for remote-monitoring of patients’ mobility.

## 1. Introduction

Advances in wearable sensor technology offers unique opportunities to clinicians and researchers to develop field-based approaches to remotely capture outcome measures traditionally studied under laboratory conditions. Remote patient monitoring with wearable sensors can be used to enhance and personalize a patient’s medical follow-up. For instance, remote monitoring of a patient’s mobility and physical functioning may enable early detection of symptoms related to specific neurological disorders, permitting rapid and personalized intervention. Amongst those wearable sensors, inertial measurement units (IMU) stand out as a promising option for remote patient monitoring. An IMU is a platform that typically incorporates accelerometers, gyroscopes and magnetometers to measure linear acceleration, angular velocity and magnetic field, respectively. Combined with a fusion algorithm, the IMU becomes an attitude and heading reference system (AHRS) that estimates the orientation of the platform in a global reference frame based on gravity and magnetic north. The use of multiple AHRS on patients, therefore, enables tracking of the patients’ joints’ motion in different contexts of activities. AHRS can be categorized as movement monitor as it allows the tracking of the quality of the motion performed over and above the quantity of motion completed [1]. The diversity of sensors used within movement monitors allows the accurate capturing of spatiotemporal gait and turn characteristics, as well as joint kinematics under specific conditions [1,2,3,4,5]. Such characteristics were proven sensitive enough to differentiate between populations under controlled conditions and were also recently used as a clinical outcome to measure the efficiency of rivastigmine during a phase-2 drug trial [4,6,7]. In-home remote monitoring of turning was also efficiently characterized using spatiotemporal features of turning allowing early identification of symptoms related to Parkinson’s disease and risk of falling [3,5]. Clinical outcomes directly based on joint kinematics using movement monitors is, however, less common. A possible explanation for this is the fact that studies have shown that the accuracy of joint orientation estimated with AHRS may vary according to the environment, the motion performed, the joint tracked, the sensor placement and the body calibration procedures and definition [2,8,9,10,11,12,13,14,15,16,17,18,19,20,21,22,23,24,25,26,27,28,29]. Indeed, AHRS uses magnetometers to complete the 3D orientation estimation of the module. Perturbations in the magnetic environment around the module can affect the ability of the algorithm to differentiate between actual motion of the platform and a change in environment. Magnetic compensation algorithms were developed to overcome those problems and are proven to work well in the case of transient perturbations (i.e., temporary perturbation of the magnetic field) [25,29,30,31,32]. However, persistent perturbations around a module will eventually affect the definition of its inertial frame. A persistent difference in the environments around two modules will affect their ability to refer to the same inertial frame, which is the foundation of AHRS’ ability to track joint kinematics. Studies have also shown, under controlled and human conditions, that the type, the direction and the velocity of the motion performed, as well as the distance of the sensors from the centre of rotation, all contribute to the orientation accuracy behaviour [9,12,13,26,33]. Indeed, the nature of the motion itself and how it is measured will have an impact on the extent of acceleration and angular velocity the AHRS is subjected to and, hence, on the accuracy of the orientation estimation. To limit the negative impacts of those factors on accuracy, some researchers have worked on more advanced orientation estimation algorithms, while others adjust the tuning of the algorithms to the movement performed, the joint tracked, and/or the population studied [24,32,34]. However, in a remote monitoring context, where a variety of movements will be performed at different speeds and in different environments throughout the day, such a specific tuning approach does not seem applicable [35]. Considering the tremendous amount of data recorded in a remote monitoring framework, several questions arise: do we need to have an optimal measurement for each and every task performed and joint tracked or do we need to know when that measurement is good or not? Building on the hypothesis that the quality of the orientation estimate depends upon the environment as well as the characteristics of the movement performed being within the optimal range of operation of the system or not, is it possible to discriminate between *good* and *bad* orientation estimates based solely on the IMU raw signals?

Artificial intelligence is a field of study focussing on the development of algorithms providing systems with an intelligent behaviour (e.g., decision-making, classification). Among those algorithms, artificial neural networks (ANNs) are recognized as an effective tool to distinguish patterns, enabling automatic classification of items based on a set of characteristics or input features [36]. The current study aims at investigating the use of a quality control (QC) algorithm, here, an artificial neural network, to automatically provide feedback on the quality of IMU joint orientation data acquired during a multitude of tasks, without further knowledge of the task performed or the joint tracked. Specifically, this paper aims at (1) developing a simple set of features based on IMU raw signals to characterize data segments; (2) verifying the ability of this set of features to discriminate between *good* and *bad* joint orientation estimates when fed into an ANN; and (3) evaluating the impact of such autonomous QC and clean-up processes on joint orientation estimate accuracy in a variety of tasks.

## 2. Materials and Methods

**Figure 1:**
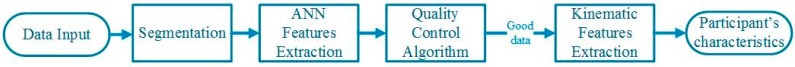
Joint orientation estimates quality control process overview.

The goal pursued by the current QC process is to discriminate *good* and *bad* sequences in regards to joint orientation estimates. Guidelines on the reliability of kinematic parameters in clinical biomechanics suggest that an error of less than 2° is widely acceptable in a context of gait analysis, as it is within the natural variation of a kinematic parameter, while data with an error between 2° and 5° are also likely to be considered acceptable, depending on the intent (e.g., rehab progress or surgical decision) [37]. Based on those guidelines, a *good* sequence was defined as a sequence with a root-mean-squared difference (RMSD) smaller than 5° from an established gold standard. Sequences with RMSD larger than 10° are considered *bad* as the accuracy of those sequences is such that the conclusion one may draw from that data may be altered by the accuracy. Finally, sequences with RMSD between 5° and 10° are considered *tolerable* in a context of in-home mobility tracking, since useful information can still be drawn from those sequences (e.g., automatic task segmentation), although direct interpretation of those sequences shall be made with care. For this study, RMSD was evaluated comparing the joint orientation estimates provided by the IMU system to an optical motion capture gold standard [38]. The general flow of the QC process proposed in this study is illustrated in Figure 1.

### 2.1. ANN Features

The quality feedback on joint orientation estimates was based on a series of features computed from the IMU sensors’ raw signals (accelerometers, gyroscopes and magnetometer). No information on the task accomplished nor the joint tracked was supplied to the network in order to remain as general as possible. All features were selected based on evidence found in the literature about factors affecting AHRS orientation estimation accuracy [9,12,13,15,25,26,33]. 

The selected input features can be separated into two major categories, namely the features related to the AHRS accuracy itself (i.e., the ability of an AHRS to measure the orientation of the segment it is attached to) and the features related to the ability of two AHRS modules to work together in order to enable accurate estimation of the orientation of a joint. Within the first category, the modules’ environment first needs to be characterized to recognize if it is perturbed around each module. This was assessed using the mean deviation of the magnetic field around the module (from a reference value) as well as the variance of the magnetic field signal, which measures the stability of the magnetic field during the sequence. As mentioned earlier, the type of motion and the velocity at which the movement is performed shall also be taken into consideration. These facets of accuracy relate directly to the level of acceleration and angular velocities the modules are subjected to and their measures were, therefore, direct inputs to the QC algorithm. The positioning of the module is also reported affecting accuracy (i.e., the segment it is attached to and/or the distance from the joint’s centre of rotation). Considering the positioning of the AHRS may vary even for the same segment (between individuals because of different anthropometric characteristics and between days for the same individual) and that the nature of the motion modulates the extent of the impact of position, this study specifically decided to use the measured AHRS’ conditions of operation to directly feed the QC algorithm in order to try to recognize situations or zones of operation that are the most at risk to produce inaccurate orientation results. It was, therefore, presumed that feeding the QC algorithm with information on acceleration and angular velocity signals would automatically take into account variations due to the positioning of the module. Finally, the direction of the motion also being reported as affecting orientation estimation accuracy, the proportion of the angular velocity measured by each axis was fed into the QC algorithm as an approximation of how the movement was distributed on the different axes (i.e., proxy of the direction of the recorded movement). 

The second category of features relates to the ability of two AHRS to work together to estimate joint orientation. The basic features chosen to be part of this category also relate to the environment. However, in this case, the focus was on the relationship between the modules applied in the joint orientation computation: are both modules referring to the same inertial reference frame? As mentioned earlier, a persistent difference in the magnetic environments around two modules will modify their reference regarding magnetic north which, in turn, will affect their respective definition of their inertial reference frame. Unfortunately, the consistency in the definition of the inertial frame between the modules is the foundation of AHRS’ ability to track joint kinematics. Thus, this relationship was first characterized using the difference in the mean magnetic fields around the two modules. A temporal component to that relationship is also believed to affect the quality of the orientation data. The difference in the mean magnetic fields from the previous sequence was, therefore, also fed into the QC algorithm for a grand total of 16 inputs, as summed up in Table 1. Inputs were normalized using the square root function and expressed as z-scores. Data with a z-score greater than ±3 standard deviations were considered outliers and brought back to this ±3 standard deviation limit where applicable. 

### 2.2. Quality Control Algorithm

**Figure 2:**
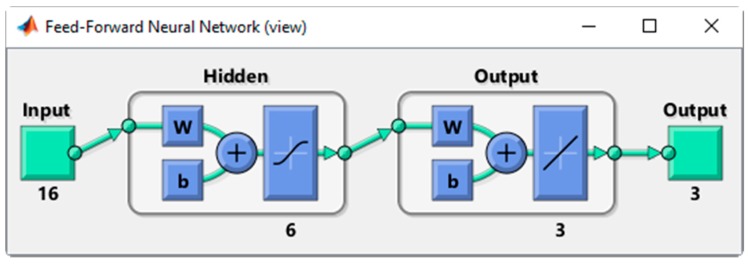
Artificial neural network architecture. The 16 inputs correspond to the features based on raw inertial signals defined in Table 1. Features are then processed at the hidden layer level composed of six neurons. Weights and bias are attributed for all inputs during the training process and neuron are activated following a symmetric sigmoid function. The resulting activation patterns are then again adjusted (output layer’s weights and bias) and summed up to determine the final classification.

An artificial neural network (ANN) approach was chosen as a proof of concept to formulate a QC algorithm capable of discriminating the *good* from the *bad* sequences with regards to orientation estimation (i.e., classify the sequences). The developed ANN is a feedforward network with a single hidden layer composed of six neurons. This simple configuration was chosen to minimize the chances of being overfit to the training sample while being complex enough to enable the detection of the subtle relationships between the different inputs. The neurons’ activation function is a symmetric sigmoid. The resulting ANN was developed in Matlab (MathWorks, Natick, MA, USA) and is shown in Figure 2. The network was trained with a Bayesian backpropagation principle and a sum of squared error (SSE) performance function. Distribution of the type of trials used for training (*good*, *tolerable*, and *bad*) was balanced using adaptable error gains (i.e., a classification error on a *bad* trial was “worth” more than on a *good* trial as there were fewer *bad* trials).

### 2.3. Experimental Procedure

**Figure 3:**
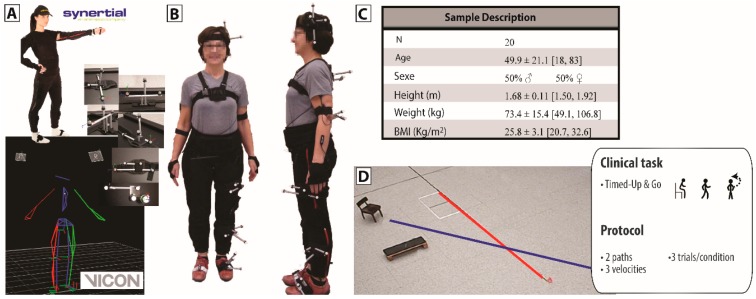
Setup and protocol. Joint accuracy validation is accomplished by comparison of the orientation data estimated by the AHRS with those obtained from an optical motion capture gold standard, VICON. (**A**) A subset of the AHRS is solidly affixed to a rigid body created with a minimum of four optical markers; (**B**) The assembled bundles are then placed on the body segments targeted for evaluation, namely the head, the upper trunk, the pelvis, and the left lower limb (thigh, shank, foot); (**C**) Twenty participants with a variety of anthropometric characteristics participated in this study, ensuring diverse conditions of realization of the tasks; (**D**) Participants were asked to perform a 5 m standardized timed-up and go (TUG), a recognized clinical test including a number of basic mobility tasks. Tests were performed along two different paths and at different velocities.

Twenty asymptomatic adults aged between 18 and 83 years old (mean age = 49.9 years old) participated in the study. Detailed characteristics of the sample are available in panel C of Figure 3. After giving their informed consent, participants were instrumented with bundles on which an AHRS (model OSv3 from Inertial Labs, Paeonian Springs, VA, USA) and a set of passive markers were solidly affixed, as shown in panels A and B of Figure 3. The bundles were attached to their dedicated limb using Velcro straps so to minimize artefacts due to skin, tissue and muscle although such issue is not of a direct concern in the present accuracy study. The chosen configuration allowed for simultaneous tracking of the joint kinematics (trunk, hip, knee, and ankle) by the AHRS, as well as by a 12 camera optical motion capture system (8 MX20, 4 T40 from VICON, Oxford, UK). Participants were asked to perform a 5 m timed-up and go (TUG) along two different paths and at different speeds (natural, slower, fastest yet safe). Each condition was repeated three times for a total of nine trials per individual. In-context accuracy assessment of the gold standard was performed for this specific protocol and determined to be 0.002° ± 0.399°, following the dynamic evaluation process described in [38].

For the purpose of the current study, data were manually segmented in Nexus (v1.8.3); therefore, using gold standard data to identify the initial sitting phase, the sit-to-stand transfer phase, the two walking segments, the turning phase, and finally the turn-to-sit transfer phase. AHRS data, originally collected at 60 Hz, were first resampled at 100 Hz to match VICON’s frequency and then synchronized using a cross-correlation approach. In both cases, joint orientation was computed as the relative orientation between two modules placed on adjacent segments, expressed in terms of their initial relative orientation. Global angular motion undergone by a joint during a specific task is then derived using the quaternion representation. Accuracy was established comparing the change in global joint motion measured by the AHRS compared to the change in global joint motion measured by the optical gold standard. Use of global motion approach to characterize accuracy is not the most common in biomechanics as it does not allow a direct association of the movement with a specific plane of motion. However, it has the advantage of focussing on the measurement accuracy assessment or in-context technological measurement accuracy assessment, ignoring errors due to alignment protocols and/or biomechanical models. Further details on the methodology of the experiment are available in our previous studies [38].

**Figure 4:**
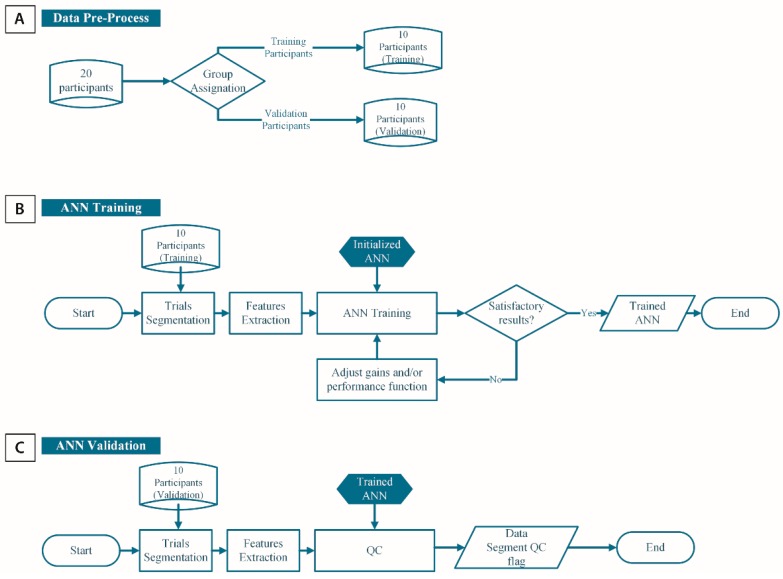
Data processing overall workflow using an ANN for joint orientation estimates quality control. (**A**) The 20 participants enrolled in the study were first divided into two groups, the first 10 being dedicated to the training of the QC algorithm while the other 10 allowed for validation of the algorithm; (**B**) Trials performed by the participants belonging to the training group were segmented into low-level tasks (sitting, sit-to-stand transfer, walking, turning, and turn-to-sit). For each data segment, a set of features based on the IMU raw signals were extracted. The ANN was then trained as long as satisfactory results regarding sensitivity and specificity are achieved and the resulting ANN becomes the so-called QC algorithm; (**C**) The performance of the QC algorithm is then verified using trials from another set of 10 participants (i.e., validation group).

A subset of 10 participants was randomly selected to train the QC algorithm (10 participants × 9 trials × 6 phases × 4 joints = 2160 data) while data gathered from the 10 other participants were used to validate the algorithm. An overall view of the data processing workflow is available in Figure 4.

### 2.4. Outcome Measures

The performance of the QC algorithm was evaluated by characterizing its overall sensitivity and specificity. In the current context, sensitivity is defined as the ability of the ANN to accept trials showing a RMSD smaller than 5° (i.e., *good* trials) while specificity relates to the network’s ability to reject *bad* trials, namely those with a RMSD greater than 10°. A trial is considered to be “accepted” if categorized as *good* by the ANN. Hence, sensitivity corresponds to the proportion of good trials that were actually identified as such by the ANN (i.e., N_accepted that are actually good_/N_good_), while specificity corresponds to the proportion of *bad* trials that were not accepted (i.e., N_bad_-N_accepted even though bad_/N_bad_). Arbitrarily, 80% was determined as an acceptable level of sensitivity and specificity to justify the interest in developing and using a neural network approach for autonomous data QC. The ultimate usefulness of the ANN relates to its ability to improve overall accuracy of the system, minimizing the variations in accuracy within and between tasks and joints reported in the literature. Hence, the usefulness of the proposed data QC approach will also be evaluated at this level, comparing accuracy levels with, and without QC, per task and joint.

## 3. Results

**Figure 5:**
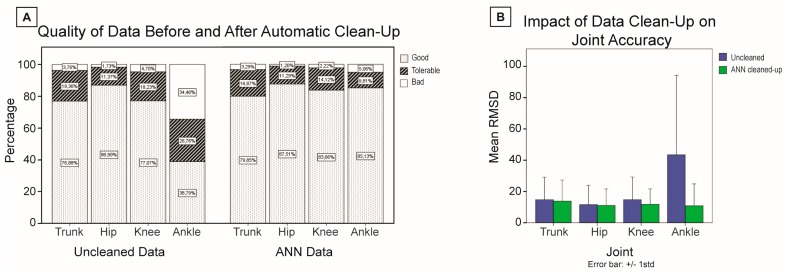
Effect of data quality control using a neural network approach on (**A**) quality of sequence distribution and (**B**) joint orientation accuracy for a diversity of tasks.

The sensitivity of the ANN was above 85% throughout the training and the validation process while its sensitivity remained above 83% (training sensitivity: 85.2%, training specificity: 84.1%; validation sensitivity: 86.7%, validation specificity: 83.6%; global sensitivity: 86.0%, global specificity: 83.9%). Data were kept when classified as *good* trials. The resulting distribution of the preserved sequences (i.e., % of good, tolerable, and bad sequences) is shown in the panel A of Figure 5 together with the original distribution of the data. The quality control process brought the distribution of the data close to equivalent for all joints. The largest difference in pre-/post-QC distribution occurs at the ankle level (original data: 38.8% *good*, 26.8% *tolerable*, 34.5% *bad*; quality-controlled data: 85.1% *good*, 9.8% *tolerable*, 5.1% *bad*). This benefit is also exposed in panel B of Figure 5, where the ankle mean RMSD is shown to decrease from 13.8° ± 12.4° to 2.8° ± 3.9°, while the trunk, the knee, and the hip showed slight improvement.

Table 2 reports the impact of the data quality control per task performed and joint tracked. The positive impact on ankle accuracy is also noticeable per task with an improvement in mean accuracy varying between 2.0° and 9.2°, and a greatly reduced dispersion.

## 4. Discussion

This study aimed at investigating the use of a quality control algorithm to provide feedback on the quality of joint orientation data estimated with AHRS. A single ANN approach for all joints and tasks was selected on the hypothesis that it will be more robust to variations in the execution of the tasks and environments when used on in-home remote monitoring data. The level of sensitivity and specificity obtained using an artificial neural network reveals that simple input features based solely on raw inertial signals (i.e., without further knowledge of the task accomplished or the joint tracked) is sufficient to identify *good* and *bad* sequences, leading to an improved accuracy for all joints. The direct impact of the QC process is also noticeable per task where the improvement in accuracy is perceptible not only in mean RMSD, but also foremost in the reduction of the dispersion of the data. As expected, most of the QC involved orientation data computed at the knee and ankle levels. In the literature, it is shown that accuracy of joint orientation decreases with increased movement velocity (above a certain level) [26]. Assuming that IMUs are optimized for a specific range of operation, robustness of the joint accuracy estimate will more likely be affected for the knee and the ankle when walking and turning at high speed. Similarly, perturbations in the environment will have a major impact on joint accuracy at slow speed as the algorithm will then be tempted, after a certain period of time, to adapt to the actual environment. In this specific experimental study, the environmental impact was more inclined to occur during the sitting and the sit-to-stand transfer phases, at the ankle and the knee level, as magnetic perturbations mainly came from the floor. Hence, a combined effect of velocity and environment explains that QC had the most impact on the knee and the ankle orientation estimates.

One of the drawbacks of such a quality feedback approach is the resulting loss of data. Although not the direct scope of this study, it should be noted that the remaining numbers of data for the ankle during walking and turning are very small. In these specific conditions, only 10% to 15% of the original data were actually good data. Combined with the loss of data due to the sensitivity/specificity of the ANN, therefore, left only a small number of good data for these conditions. Improvement of the input features could enhance the sensitivity/specificity of the ANN and, therefore, reduce the loss of data occurring at the ankle level; however, improvement in the fusion algorithm for this specific joint would be even more desirable. For a specific case which would bring similar results at the ankle level, gait and turn mobility characteristics at the ankle level should better rely on raw inertial signals or consider correction of the orientation data first [39].

Again, the simple input features selected were enough to demonstrate that sensors’ raw signals encompass the required information to predict the quality of the joint orientation estimation, but an improvement in these features may enhance differentiation between *good* and *tolerable* sequences. More complex features could also improve the classification of “extreme” sequences, namely those sequences where magnetic perturbation is coupled with high-speed movement (e.g., ankle during fast walking). The current version of the ANN allowed good QC specificity to *bad* trials in this situation, but the sensitivity to *good* sequences could be further improved. For example, the use of the maximum angular velocity (instead of the mean) could possibly enhance the response of the ANN for the ankle. Furthermore, the consistency in the sensitivity and specificity level attained during training and validation phases also tends to prove that the QC approach based on features extracted from IMU raw inertial signals is independent of the participants’ characteristics, as these two actions were performed on a different set of participants. Furthermore, both young and elderly subgroups were composed of healthy participants and conditions in the realization of the tasks varied within a subgroup (e.g., participants’ walking speed varied between individuals, some elderly participants walked faster than younger participants). Hence, in this case, variations in the conditions of motion is mainly attributed to anthropometric characteristics rather than age. 

A limit to this study is the fact that the developed QC algorithm was only tested using one type of AHRS. One could, therefore, wonder if the approach is transferable to another system. Based on the conclusions of a study comparing three different commercially-available AHRS, factors affecting the robustness of AHRS orientation data accuracy (i.e., velocity, direction of motion, and environment) are consistent throughout the systems although the extent of the effects varies among the companies [12,13]. Hence, such a quality control approach may have to be retrained when used with different AHRS, but the principle would still work. 

Finally, the current study methodology segmented the trials per task and applied the QC process on the created segments of data. However, it would be interesting to investigate the possibility of using such a QC process on a temporal basis (e.g., using 20s windows). The temporal impact of magnetic perturbations could, therefore, be better characterized. On the other hand, using a task segmentation approach improves the straight-forward usability of the QC process concept. Combined with automatic task segmentation algorithms based on AHRS data for free-living environments, the entire QC process introduced in Figure 1 could be autonomously performed [35,40,41]. Indeed, this work will eventually allow to automatically segment complex free-living activities and assess the performance of the person tested, while providing QC on the recorded data.

## 5. Conclusions

This study confirms the hypothesis that it is possible to perform an autonomous quality control on joint orientation data estimated from inertial signals. With the input features selected, being based solely on the inertial signals, the approach is generalizable to all joints and tasks, as shown in the current experiment. Autonomous QC post-processing of joint orientation data may be of particular interest for remote-monitoring of patients’ mobility, enabling the use of orientation data on top of raw inertial signals.

## Figures and Tables

**Table 1:** QC Input Features.

Features Category	Aspect	Input Feature
*AHRS accuracy*	Environment	Deviation of the mean magnetic field from a reference value, for each module (inputs 1–2) Variance of the magnetic field signal around each module (inputs 3–4)
	Motion performed	Mean acceleration per module (inputs 5–6) Mean angular velocity per module (inputs 7–8)
	Direction of motion	Proportion of angular velocity measured on each axis (inputs 9–11 for module 1, inputs 12–14 for module 2)
*Joint estimation accuracy*	Environment	Difference between the two modules’ magnetic field (input 15)
		Difference between the two modules’ magnetic field in the previous sequence (input 16)

**Table 2:** Impact of autonomous quality control of orientation data sequences per task and joint.

		Original Data			Cleaned-up Data (ANN)	
		N_total_	N_good_	RMSD_total_	N_accepted_	RMSD_accepted_
**Sit**	*Trunk*	180	177	0.9° (1.4°)	177	0.7° (0.6°)
	*Hip*	180	177	0.7° (2.5°)	178	0.5° (0.6°)
	*Knee*	180	177	0.9° (2.8°)	178	0.7° (1.0°)
	*Ankle*	177	159	3.4° (10.1°)	157	1.2° (2.2°)
**STS**	*Trunk*	175	120	4.2° (2.5°)	113	3.6° (2.0°)
	*Hip*	175	165	2.1° (1.9°)	177	2.1° (1.9°)
	*Knee*	180	167	2.4° (2.3°)	169	2.2° (1.7°)
	*Ankle*	176	122	5.8° (9.4°)	124	3.8° (3.7°)
**Walk**	*Trunk*	349	266	4.3° (4.5°)	325	4.2° (4.0°)
	*Hip*	349	301	3.5° (4.0°)	331	3.3° (3.3°)
	*Knee*	359	260	4.3° (2.5°)	275	4.2° (2.4°)
	*Ankle*	351	51	14.3° (12.4°)	7	5.1° (2.4°)
**Turn**	*Trunk*	166	117	4.1° (2.5°)	141	4.0° (2.3°)
	*Hip*	166	133	3.8° (1.8°)	161	3.8° (1.9°)
	*Knee*	180	108	4.7° (2.6°)	81	4.5° (2.3°)
	*Ankle*	176	18	15.2° (10.9°)	2	6.4° (0.2°)
**Turn-to-sit**	*Trunk*	168	118	4.4° (3.5°)	157	4.5° (3.6°)
	*Hip*	168	126	3.9° (2.0°)	161	3.8° (1.9°)
	*Knee*	165	108	5.6° (5.7°)	62	4.1° (2.5°)
	*Ankle*	159	53	12.5° (15.2°)	26	6.0° (4.5°)

Note: N_total_—number of data; N_good_—number of good data (i.e., RMSD ≤ 5°); RMSD_total_—Mean (std dev.) root-mean-square difference between AHRS joint orientation and reference joint orientation (all data); N_kept_—number of data classified as good by the ANN; RMSD_accepted_—Mean (std dev.) root-mean-square difference between AHRS joint orientation accepted by the ANN and their reference joint orientation.

## Abbreviations

The following abbreviations are used in this manuscript:

AHRS	Attitude and Heading Reference System
ANN	Artificial Neural Network
IMU	Inertial Measurement Unit
